# Formation of Fe-Te Nanostructures during *in Situ* Fe Heavy Doping of Bi_2_Te_3_

**DOI:** 10.3390/nano9050782

**Published:** 2019-05-22

**Authors:** Jing Liang, Xiong Yao, Yu Jun Zhang, Fei Chen, Yuanzhen Chen, Iam Keong Sou

**Affiliations:** 1 Department of Physics, Hong Kong University of Science and Technology, Hong Kong 999077, China; jliangah@connect.ust.hk (J.L.); xy226@physics.rutgers.edu (X.Y.); 2 William Mong Institute of Nano Science and Technology, Hong Kong University of Science and Technology, Hong Kong 999077, China; 3 Department of Physics, Southern University of Science and Technology of China, Shenzhen 518055, China; 11648009@mail.sustc.edu.cn (Y.J.Z.); chenyz@sustc.edu.cn (Y.C.); 4 National Laboratory of Solid State Microstructures, School of Physics, and Collaborative Innovation Center of Advanced Microstructures, Nanjing University, Nanjing 210093, China; cf19870924@163.com; 5 Institute for Quantum Science and Engineering, Southern University of Science and Technology of China, Shenzhen 518055, China

**Keywords:** MBE growth, doping with monotonically increasing concentration, Bi_2_Te_3_ thin film, Fe-Te nanostructures, interfacial superconductivity

## Abstract

To study the *in situ* doping effect upon monotonically increasing dopant concentration, a Bi_2_Te_3_ layer doped with Fe up to ~6.9% along the growth direction was fabricated by the molecular beam epitaxy (MBE) technique. Its resistance versus temperature curve displays a superconductivity transition at about 12.3 K. Detailed structural and chemical analysis via X-ray diffraction (XRD), scanning electron microscopy (SEM), transmission electron microscopy (TEM), and energy-dispersive X-ray spectroscopy (EDS) reveal that this layer consists of two types of unexpected Fe-Te nanostructures: one is FeTe thin layer formed near the surface, and the other is FeTe_2_ nanorod embedded in the Bi_2_Te_3_ layer. Based on the results of further electrical and magnetotransport studies, it is likely that the observed superconductivity originates from the interface between the FeTe nanostructure and the neighboring Bi_2_Te_3_ layer. We have addressed the formation mechanisms of the observed nanostructures, which is attributed to the strong reaction between Fe and Te atoms during the growth process. The findings of this study also provide an unusual approach to synthesizing nanostructures via heavy doping if the dopant element is strongly reactive with an element in the host matrix.

## 1. Introduction

Three-dimensional (3D) topological insulators (TIs) are a new class of materials that have attracted great interests in both theoretic [1,2,3,4] and experimental [5,6,7] researches in recent years. In 3D TIs, there is an insulating gap in bulk states accompanying with robust metallic topological surface states (TSSs) arising from band inversion caused by strong spin–orbit coupling. These TSSs are protected by time-reversal symmetry (TRS) and consist of spin-momentum locked massless Dirac electrons. The most widely studied 3D TIs are A_2_B_3_-family including Bi_2_Se_3_, Bi_2_Te_3_, and Sb_2_Te_3_, which have larger band gaps as well as simpler surface structure (only single Dirac cone in their surface states) compared to the first experimentally observed 3D TI, the Bi_1−x_Sb_x_ alloy.

The doping of TIs is one of the promising ways to realize new types of devices and new classes of materials. For instance, induced superconductivity (SC) has been reported in Bi_2_Se_3_ with intercalating Cu or Sr atoms between neighboring quintuple layers [8,9,10,11,12]. A theoretical study predicts that topological SC in doped topological insulators may be suitable for hosting Majorana fermions and have potential application in fault-tolerant topological quantum computing [13]. For Bi_2_Se_3_ with intercalating Cu dopant, it was showed that the spin-polarized TSSs are preserved at the Fermi level while SC occurs in bulk regime, suggesting that superconducting Cu_x_Bi_2_Se_3_ may be suitable for trapping non-Abelian Majorana fermions [14]. Equally interesting, quantum anomalous Hall (QAH) effect is expected to emerge in magnetically doped TIs due to the broken TRS [15]. In fact, quantization of the Hall resistance of h/e^2^ at zero field, a signature of QAH effect, was observed in Cr-doped (Bi,Sb)_2_Te_3_ thin films at temperature below 30mK [16,17]. The electrical and magnetic properties of TIs are also found to be sensitive to extrinsic chemical doping. It has been reported that Fe-substituted Bi_2_Se_3_ favors ferromagnetic interactions while antiferromagnetic interactions dominates in Cr-substituted Bi_2_Se_3_ [18]. N. H. Jo et al. reported that the incorporation of Fe in bulk single crystal of Bi_2-x_Fe_x_Te_3_ with *x* ranging from 0.08 to 0.3 leads to the change of the conduction type from n-type to p-type at *x* = 0.3 (equivalent to 6% for the atomic concentration of Fe) [19].

In 2014, our group discovered a novel two-dimensional (2D) SC at the interface of a Bi_2_Te_3_/FeTe heterostructure [20]. Another independent study also observed a superconducting energy gap by spin-polarized scanning tunneling spectroscopy for one unit cell FeTe on Bi_2_Te_3_ substrate with *T_C_* = 6 K [21]. However, the underlying mechanism of this SC has not been understood so far. In this study, we fabricated a Bi_2_Te_3_:Fe sample with varying Fe concentration along the growth direction so as to study if a certain Fe doping concentration in Bi_2_Te_3_ could make it superconducting, which is based on the thought that the observed interfacial SC at the Bi_2_Te_3_/FeTe heterostructure may be caused by forming a superconducting Bi_2_Te_3_:Fe layer at the interface of the heterostructure at a certain doping level due to Fe diffusion. Interestingly, this sample indeed shows a superconducting transition at its resistance vs temperature curve. We address the cause of the observed SC and the formation of two types of Fe-based nanostructures found in this sample through an unexpected formation mechanism, attributed to the non-thermal equilibrium growth mode of the molecular beam epitaxy (MBE) technique. It is worth pointing out that usually nanostructures can be fabricated using either top-down or bottom-up approach. In this study, we present a special approach, based on *in situ* heavy doping, to synthesizing nanostructures under the condition that the dopant element is strongly reactive with an element in the host matrix.

Our studies reveal that *in situ* doping of a highly reactive dopant using a non-thermal equilibrium growth technique could result in unexpected phases of nanostructures embedded in the host matrix, providing a new path for forming new nanostructured materials.

## 2. Materials and Methods

All samples studied in this work are fabricated in a VG-V80H MBE system (VG SCIENTIFIC, Waltham, MA, USA) equipped with *in situ* reflection high-energy electron diffraction facility. High purity Bi_2_Te_3_ compound source (GoodFellow, Huntingdon, UK; 99.999%) together with an Fe elemental source (GoodFellow, Huntingdon, UK; 99.95%) were used for the MBE growth. The key sample for our studies is a multilayer sample. Prior to its growth, a semi-insulating GaAs (111)B substrate (AXT Inc., Fremont, CA, USA) was first thermally heated to 580 °C to remove the passivation oxide. Then a pure Bi_2_Te_3_ thin film of thickness ~15 nm was grown, followed by the growth of a set of Bi_2_Te_3_ layers with gradually increasing Fe concentration by opening the shutter of the Fe effusion cell with its cell temperature varying from a starting value of 880 °C to an ending value of 1150 °C with a step of 30 °C; the nominal thickness of each layer is estimated to be ~10 nm. During the whole growth process, the temperatures of the substrate and the Bi_2_Te_3_ effusion cell were kept at 235 °C and 420 °C, respectively. We have made an estimation of the highest apparent doping concentration of Fe in this sample with a result of ~8% in atomic concentration, which was estimated based on the growth rates of a pure Bi_2_Te_3_ layer and a pure FeTe layer grown with the Fe cell temperature at 1150 °C assuming the sticking coefficient of Fe in both the growth of Bi_2_Te_3_:Fe and FeTe is equal to one. A comparison group of samples containing a pure 24 nm Bi_2_Te_3_ layer grown on a GaAs (111)B (AXT Inc., Fremont, CA, USA) substrate and a pure 150 nm FeTe layer grown on a ZnSe(~70 nm)/GaAs(100) substrate (AXT Inc., Fremont, CA, USA) were also fabricated. ZnSe compound source (Alfa Aesar, Ward Hill, MA, USA; 99.999%), Te elemental source (ESPI Metals, Ashland, OR, USA; 99.999%), Bi_2_Te_3_ and Fe sources were used for the growth of these samples.

Each sample was cut into long strips (with dimension ~2 × 6 mm^2^), and conventional four-point electric contacts were made on the surface using silver paint as the contact material for conducting transport measurements. Their electrical and magnetotransport properties were measured in a Quantum Design physical property measurement system (PPMS, model 6000, Quantum Design, Inc., San Diego, CA, USA). High-resolution X-ray diffraction (HRXRD) measurements were conducted by PANalytical multipurpose diffractometer with an X’celerator detector (PANalytical X’Pert Pro, Malvern, UK) for composition and crystalline phase characterizations. Cross-sectional high-resolution transmission electron microscopy (HRTEM) images were taken in a JEOL 2010F TEM (JEOL Ltd., Tokyo, Japan) with acceleration voltage of 200 kV in conventional TEM mode. Plan view scanning electron microscopy (SEM) images were taken in a JEOL JSM-6390 SEM (JEOL Ltd., Tokyo, Japan). Both TEM and SEM systems are equipped with energy dispersive X-ray spectroscopy (EDS). The scanning TEM (STEM) studies were performed using an aberration-corrected JEOL JEM-ARM200F TEM (JEOL Ltd., Tokyo, Japan) working under dark-field, which provides both EDS mapping and corresponding TEM imaging.

## 3. Results and Discussion

**Figure 1:**
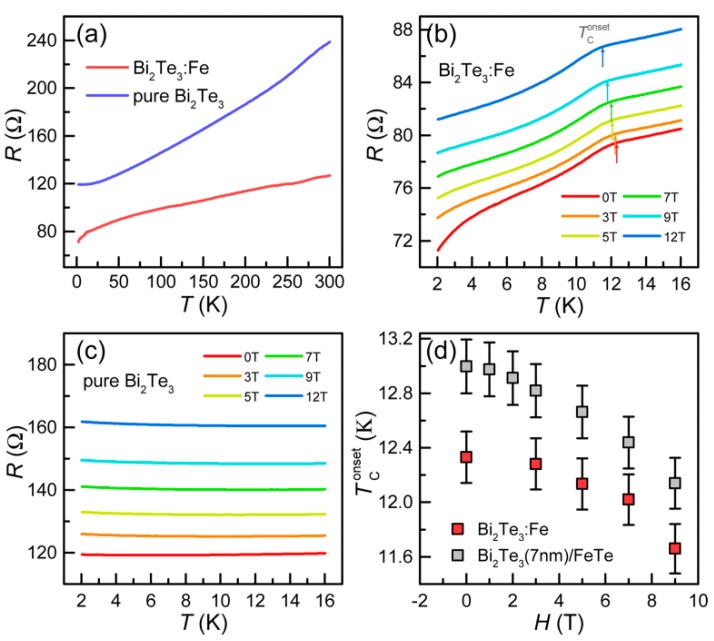
Temperature-dependent transport and magnetotransport properties. (**a**) Temperature dependence of the resistance of a pure Bi_2_Te_3_ thin film and the Bi_2_Te_3_:Fe thin film. (**b**) Temperature-dependent resistance of the Bi_2_Te_3_:Fe thin film under magnetic fields ranging from 0 to 12 T. $T_{C}^{onset}$ values at which the resistance starts to show a sudden drop are marked by arrows. (**c**) Temperature-dependent resistance of the pure Bi_2_Te_3_ thin film, showing no evidence of SC. (**d**) Magnetic field dependent $T_{C}^{onset}$ for both the Bi_2_Te_3_:Fe thin film and a Bi_2_Te_3_(7 nm)/FeTe heterostructure shows a similar trend, indicating they share the same origin of the SC.

First, we measured the electrical and magnetotransport properties of the Bi_2_Te_3_:Fe thin film and a pure Bi_2_Te_3_ thin film aiming to investigate the effects of Fe doping. Figure 1a shows the temperature dependence of the in-plane resistance of both samples. It is well known that a defect-free intrinsic Bi_2_Te_3_ thin film with thickness > 1 nm has gapped bulk states and the Fermi level lies in the energy gap (very close to the valence band) [4], thus it is expected to show a narrow-gap semiconductor behavior at low temperature. The Bi_2_Te_3_ thin film studied in this work, however, shows a metallic behavior instead as displayed in Figure 1a. This behavior is believed to be attributed to Te vacancies generated in the synthesizing process, which shifts the Fermi level into the conduction band. Generation of Te vacancies in Bi_2_Te_3_ is common in MBE growth [22] and other growth techniques [6,23]. As can be seen in Figure 1a, the *R* vs *T* curve of the Bi_2_Te_3_:Fe thin film overall displays a metallic behavior, however, a sudden drop in resistance occurs at temperature ~12.3 K at zero magnetic field. Figure 1b shows the temperature-dependent resistance of the Bi_2_Te_3_:Fe thin film in the temperature range from 2 to 16 K at the presence of external magnetic field up to 12 T, revealing that the onset of the drop of resistance (marked by arrows) shifts to lower temperature as the external magnetic field increases. Figure 1c shows the temperature-dependent resistance of the pure Bi_2_Te_3_ thin film under magnetic fields of the same range; however, apart from seeing a moderate positive magnetoresistance effect, no sign of a superconducting transition can be seen. The above experimental observations indicate that our Bi_2_Te_3_:Fe thin film enjoys a SC feature attributed to the incorporation of Fe dopants.

However, neither previous theoretical [24] nor experimental [18,19] studies on Fe uniformly doped Bi_2_Te_3_ samples predict or observe SC. Moreover, it is not likely that the SC at ~12 K shown in our Bi_2_Te_3_:Fe sample comes from an ordinarily Fe-doped Bi_2_Te_3_ thin film because the induced SC in A_2_B_3_-type topological insulators usually has a much lower *T_C_*. For example, it was reported that bulk SC can be achieved with *T_C_* of 2.28 K in Tl_0.6_Bi_2_Te_3_ [25], 2.9 K in Sr-intercalated Bi_2_Se_3_ [10,11,12], and up to 3.8 K in Cu_x_Bi_2_Se_3_ [8,9,14]. In fact, the detected SC in our Bi_2_Te_3_:Fe sample with *T_C_* of ~12 K is reminiscent of the 2D SC in the Bi_2_Te_3_/FeTe bilayer heterostructure discovered by our group previously [20], of which the maximum *T_C_* of a series of Bi_2_Te_3_/FeTe heterostructure sample with different Bi_2_Te_3_ thicknesses is also ~12 K. In order to find out the source of the observed SC, we conducted detailed structural and chemical analysis on the Bi_2_Te_3_:Fe sample.

**Figure 2:**
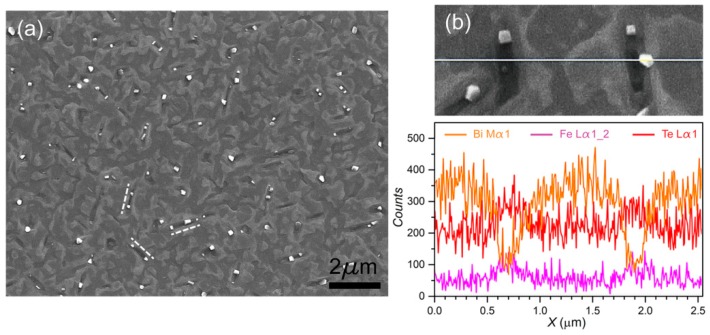
Surface morphology of the Bi_2_Te_3_:Fe thin film surface. (**a**) Plan view SEM image of Bi_2_Te_3_:Fe thin film surface, showing nanorods are embedded in the thin film and align along three preferred directions (marked by dash lines). Island formations are found on the nanorods. (**b**) A finer SEM image with a marked EDS scanning line (upper) and the corresponding EDS spectra (lower).

Figure 2a displays a plan view SEM image of the surface of the Bi_2_Te_3_:Fe thin film within an area of 11 × 15 µm^2^. It can be seen that some nanorods are embedded in the thin film with length around 1 μm and width around 200 nm. These nanorods are aligned in three directions (marked by the dash lines) and the angle between two neighboring directions is 120°. Another type of nanostructure is the islands that are favorably formed on the nanorods. To obtain the chemical composition of the nanorods and the islands, line-scanning EDS was performed across these two nanostructures as shown in Figure 2b in which the solid line indicates the trace of the focused electron beam. The corresponding EDS spectrum is depicted in the lower row of Figure 2b. It clearly shows that the X-ray signal generated by Bi atoms drops sharply and the opposite trends occur for those of Fe and Te atoms when the electron beam enters the nanorods. On the other hand, the chemical composition of the island seems to be the same as that of the neighboring Bi_2_Te_3_ region, though the signals of Bi and Te at the island site are contributed by both the island and underlying Bi_2_Te_3_ layer, however, as the thickness of the island is about half of the underlying Bi_2_Te_3_ layer (this will be addressed later), this claim should still be valid. Thus one can conclude that the nanorods likely consist of Fe and Te atoms mainly, however, it is not reliable to determine their chemical ratio by the EDS technique since it is well known that EDS is just a semiquantitative technique. HRXRD and HRTEM were then performed with the aim to achieve more quantitative structural and chemical analysis for the Bi_2_Te_3_:Fe thin film.

**Figure 3:**
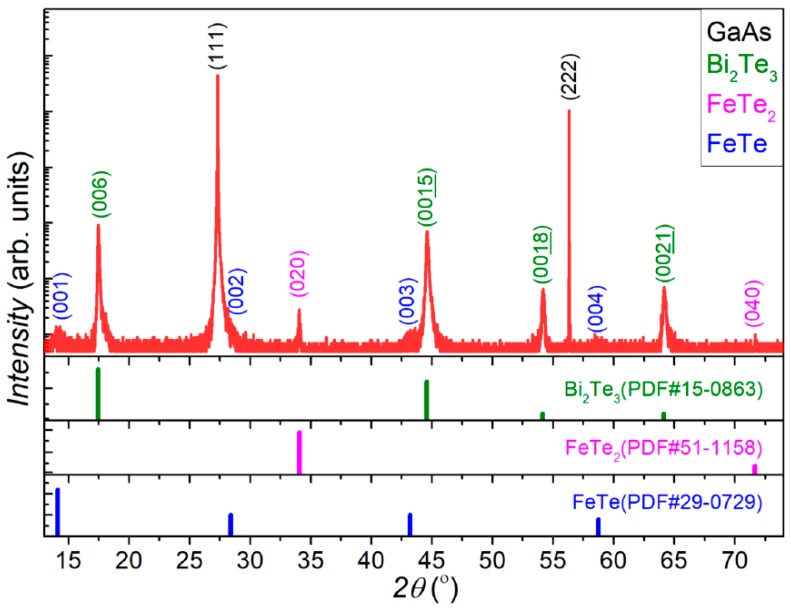
Symmetric 2*θ*-ω scan high-resolution X-ray diffraction (HRXRD) of the Bi_2_Te_3_:Fe thin film with 2*θ* ranging from 13 to 74°. The lower part shows PDFs for the three compounds contained in the thin film.

The top part of Figure 3 plots the HRXRD profile of the Bi_2_Te_3_:Fe thin film in symmetric 2*θ*-ω scan mode using an X-ray beam with wavelength λ of 1.540598 Å generated from Cu K-α1. The composition of the Bi_2_Te_3_:Fe thin film was determined through a detailed study on this HRXRD profile. The lower part of Figure 3 shows the powder diffraction files (PDFs) of the three crystalline materials contained in the thin film, where only the peaks oriented along the normal of the sample surface are extracted for clarity. As can be seen in Figure 3, the two strongest peaks are the (111) and (222) peaks of the GaAs substrate, the next four strong peaks match well with the (0 0 6), (0 0 15), (0 0 18), and (0 0 21) peaks of Bi_2_Te_3_. From these four peaks we have calculated the corresponding lattice parameter in the z-direction to be *c* = 30.45 Å, which indeed agrees well with the reported standard value of *c* = 30.48 Å [26]. The two peaks at 2*θ* of 34.06° and 71.71° match quite well with the (020) and (040) peaks of an unexpected phase, FeTe_2_. A careful inspection of Figure 3 could also find the evidence of the existence of the four characteristic diffraction peaks of (001), (002), (003), and (004) of another unexpected phase—FeTe—though the (002) peak is buried in the GaAs (111) peak. In the following two paragraphs, the solid evidence, provided by HRTEM imaging studies, for the existence of FeTe_2_ and FeTe phases in our Bi_2_Te_3_:Fe sample will be addressed.

**Figure 4:**
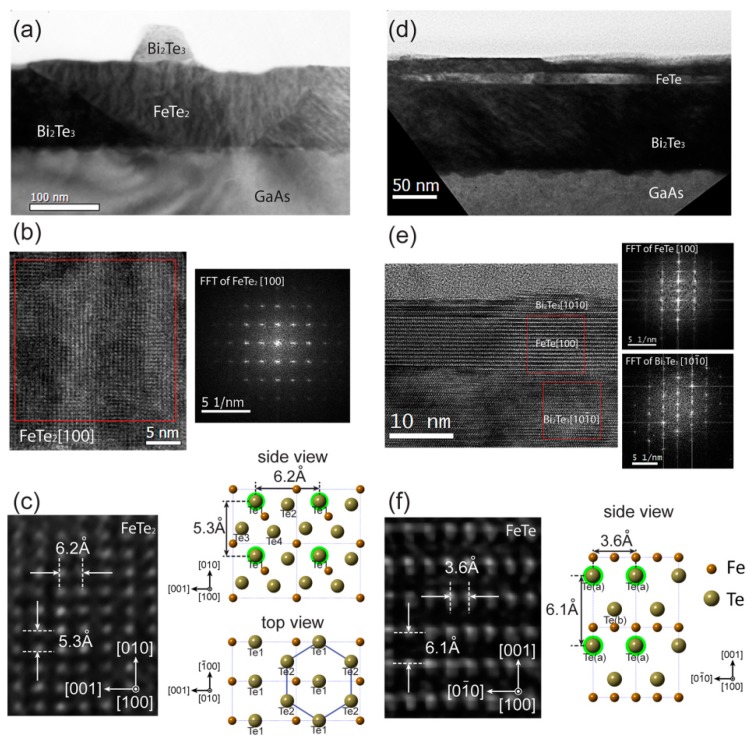
TEM images and corresponding analysis of the two types of Fe-Te nanostructures and Bi_2_Te_3_ islands. (**a**) Cross-sectional TEM image of a typical FeTe_2_ nanorod on which there is a Bi_2_Te_3_ island. (**b**) Zoomed-in HRTEM image showing the FeTe_2_ region in a and its corresponding FFT of the area enclosed in b. (**c**) HRTEM image of a FeTe_2_ nanorod and its schematic lattice in two orientations. (**d**) Cross-sectional TEM image of another type of nanostructure which is located near the surface of the sample. Chemical and structural analyses show that this type of nanostructure is FeTe. (**e**) Zoomed-in image of FeTe nanostructure and its neighboring Bi_2_Te_3_ region together with their corresponding FFT patterns. (**f**) HRTEM image of the FeTe nanostructure and its schematic lattice. It should be noted that the atoms marked with blurred green color in the top right drawing in **c** and in the right drawing in f refer to those atoms at the top lattice plane of the corresponding atomic lattice model.

Figure 4 displays the TEM images, fast Fourier-transform (FFT) patterns and relevant schematic lattice drawings for the nanostructures and thin film layer involved in the Bi_2_Te_3_:Fe sample. Figure 4a shows the cross-sectional TEM image of a typical example of the nanorods shown in the SEM image in Figure 2. As shown, it has a trapezoidal cross-section and its bottom surface reaches the top surface of the GaAs substrate. Figure S1 in Supplementary Materials shows a cross-sectional TEM image of another typical nanorod that lands its root inside the Bi_2_Te_3_ layer. The reason why we could claim that this type of nanostructure in the film is the nanorods seen in the SEM image is that their width and number density in the TEM images match quite well with those derived from the SEM image. In the left of Figure 4b, a finer HRTEM image of the nanorod shown in Figure 4a is displayed and the FFT pattern of a region of the nanorod (marked by the square in Figure 4b is shown in the right which displays a pattern match very well with a simulated diffraction pattern of FeTe_2_ along [100] zone axis. The left of Figure 4c shows the HRTEM image of a small region of the FeTe_2_ nanorod, where the dominating lattice presents a layer of Te atoms within the same lattice plane. This figure also shows the measured lattice spacings of 6.2 Å and 5.3 Å, corresponding to the horizontal and vertical values, respectively, both agree well with the corresponding lattice spacings of an atomic lattice model of FeTe_2_ shown in the upper right of Figure 4c, where the highlighted four Te1 atoms correspond to one unit cell of the image shown in the left of Figure 4c. At the lower right of Figure 4c, the top view of the FeTe_2_ lattice with its [010] direction pointing out of the page and along the c-axis of Bi2Te3, that is, normal to the sample surface, is shown. One can see that the upper two layers of Te atoms form a quasi-hexagonal shape with its lattice dimension enjoys a small lattice-mismatch of ~16% as compared with that of Bi_2_Te_3_ (001). We believe that this might be the reason why the FeTe_2_ nanorods prefer to grow upward along its [010] direction because this quasi-hexagonal shape might have provided a relatively better lattice match with the hexagonal lattice arrangement of Bi_2_Te_3_ along its *c*-axis than other possible growth directions. A related issue regarding the preferred three lateral growth directions of the FeTe_2_ nanorods as shown in the SEM image of Figure 2a, perhaps can be attributed to the well-known three-fold symmetry of the Bi_2_Te_3_ lattice planes perpendicular to its *c*-axis [4], which leads to the lateral growth of the FeTe_2_ nanorods to select the corresponding three directions that enjoy the highest symmetry. As shown in Figure 4a, an island with base width ~100 nm and height ~50 nm can be found located above the FeTe_2_ nano-rod, which corresponds to one of the many islands seen in the SEM image shown in Figure 2a. In Figure S2 of the Supplementary Materials, we have provided evidence via its HRTEM images and EDS analysis to show that the islands are Bi_2_Te_3_ in composition and likely contain several grains with different orientations.

Among the HRTEM images of the Bi_2_Te_3_:Fe sample, we have found another type of nanostructures, which can only be found near the surface of the sample and its number density is much less than that of the FeTe_2_ nanorods. In Figure S3 of the Supplementary Materials, the EDS profile performed on this nanostructure indicates that it mainly consists of Fe and Te in composition. Figure 4d shows the HRTEM image of this relatively rare nanostructure and its neighboring region. The left and right of Figure 4e show a zoomed-in HRTEM image of Figure 4d and its FFT patterns of this nanostructure and its neighboring region, respectively, with the top FFT pattern matches with the diffraction pattern expected from [010]-oriented FeTe and the bottom one matches with that of [$10\bar{1}0$]-oriented Bi_2_Te_3_. The left of Figure 4f shows an HRTEM image of the FeTe nanostructure at atomic scale, revealing the two orthogonal lattice parameters to be a = 3.6 Å and c = 6.1Å, which are in good agreement with the schematic drawing of a FeTe lattice viewed along the [100] direction [27], which is displayed in the right of Figure 4f, where the highlighted four Te(a) atoms correspond to one unit cell of the image shown in the left of Figure 4f. In fact, the existence of such a FeTe nanostructure in the Bi_2_Te_3_:Fe sample as revealed via cross-sectional HRTEM studies echoes the detection of a FeTe phase in the HRXRD profile as shown in Figure 3. Due to the time-consuming TEM sample preparation process and limited area of thin region in TEM sample, it is difficult to estimate a precise density of FeTe nanostructures. A plan view TEM sample was also examined; however, we could not observe the FeTe phase but could observe FeTe_2_ nanorods, because FeTe lies under the sample surface. Thus it is not possible to tell the shape of the FeTe nanostructures, either, at the moment. A rough estimation is that the distance between two FeTe nanostructures formed near the sample surface is ~5 μm, and the length of FeTe nanostructures ranges from 100 to 400 nm. As for FeTe_2_ nanorods, their surface density counted from SEM image is 24.7%. Assuming the surface density of FeTe_2_ nanorods is a constant within the most top 3 nm layer, and taking the volume per each element in Bi_2_Te_3_ and FeTe_2_ phases into consideration, we calculated the highest Fe atomic doping concentration at the top surface is 6.9%, which is close to the value of 8.0% that was estimated based on the growth rate.

Through the above detailed structural and chemical analysis of the various phases exist in the Bi_2_Te_3_: Fe sample, now it is clear that the observed SC at ~12 K as displayed in Figure 1 can be attributed to the heterojunction formed by the FeTe nanostructure and its neighboring Bi_2_Te_3_ layer, because such a heterojunction has been demonstrated by us previously to enjoy SC with a critical temperature ~12 K if its Bi_2_Te_3_ component is thicker than 5 nm [20]. The magnetic field dependences of the onset temperature of the detected drop of resistance for the Bi_2_Te_3_:Fe thin film, and a Bi_2_Te_3_(7 nm)/FeTe heterostructure is plotted in Figure 1d, which indeed shows a similar trend. In Figure S4 of the Supplementary Materials, we have provided further electrical and magnetotransport results, which provide further evidence that the observed SC in the Bi_2_Te_3_:Fe sample likely shares the same origin of the SC at the interface of a Bi_2_Te_3_/FeTe heterostructure.

**Figure 5:**
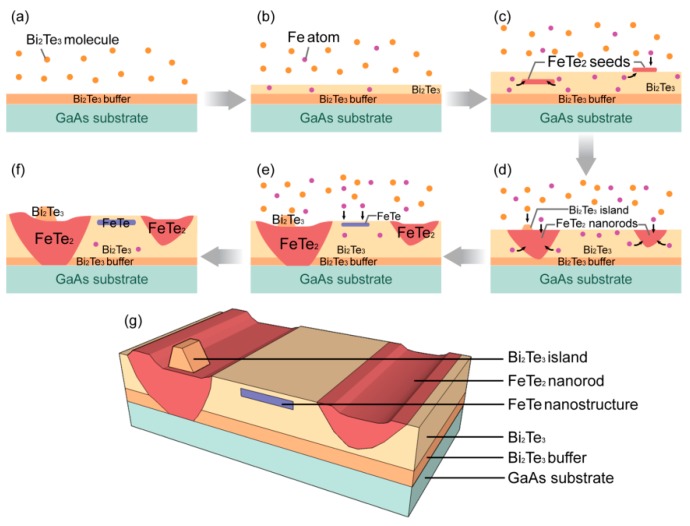
Phenomenological model for the formation of the observed nanostructures in the Bi_2_Te_3_:Fe sample. (**a**) A Bi_2_Te_3_ buffer layer was grown; (**b**) growth of Bi_2_Te_3_:Fe was initiated; (**c**) at high enough Fe flux, FeTe_2_ seeds were formed; (**d**) the seeds turned into FeTe_2_ nanorods and for the nanorods close to the Bi_2_Te_3_ buffer layer, they could extend down to the buffer layer. Bi_2_Te_3_ islands were also formed near the ends of the FeTe_2_ nanorods; (**e**) strong interaction between Fe and Te atoms resulted in the breaking of the bonds of the Bi_2_Te_3_ buffer and thus some of the FeTe_2_ nanorods initiated near the buffer layer could even reach the top surface of the GaAs substrate. Due to the lower upward growth rate of the FeTe_2_ nanorods as compared with that of the neighboring Bi_2_Te_3_ layer, the top surfaces of the nanorods appear to be dented; near the end of the growth, FeTe nanostructure was formed as the increasing Fe flux reached a certain value; (**f**) the cross-sectional drawing of the Bi_2_Te_3_:Fe sample at the end of the growth; and (**g**) a three-dimensional drawing for the as-grown Bi_2_Te_3_:Fe sample consisting of the two types of Fe-Te nanostructures and Bi_2_Te_3_ island.

In the following paragraph, we present a phenomenological model for the formation mechanisms of the three types of nanostructures, namely FeTe_2_ nanorods, FeTe nanostructure, and Bi_2_Te_3_ islands, found in the Bi_2_Te_3_: Fe sample based on the findings presented above. We believe that the formation of the first two Fe-Te nanostructures are attributed to the strong reaction between the Fe atoms with the Te atoms either from the Bi_2_Te_3_ source flux or the Te lattice atoms in the as-grown Bi_2_Te_3_ layers. The fact that in the Bi_2_Te_3_:Fe sample, a 15-nm-thick pure buffer Bi_2_Te_3_ layer was first grown before the cosupplying of both Bi_2_Te_3_ and Fe fluxes, however, most of the FeTe_2_ nanorods were found to reach the top surface of the GaAs substrate. This indicates that the strong reaction between Fe and Te atoms could even lead to the breaking of the bonds of Bi_2_Te_3_ to form a FeTe_2_ phase. Since the Bi_2_Te_3_:Fe sample was grown with an increasing Fe flux, the FeTe_2_ nanorods formed at some preferred seeds correspondingly grow in bigger size along the upward direction (normal to the sample surface), forming a trapezoidal cross-sectional shape as shown in Figure 4a,b. However, the growth rate of FeTe_2_ nanorods along the upward direction is slower than the growth rate of the neighboring Bi_2_Te_3_ layer, thus these nanorods appear to be dented as shown in the SEM image displayed in Figure 2a. As the highest Fe flux was provided near the end of the growth, it provided the condition to form a Fe-Te compound with more rich in Fe composition as compared with the FeTe_2_ nanorods, thus this explains why the early mentioned FeTe nanostructures could be formed near the surface of the sample. Since Bi_2_Te_3_ flux were also provided simultaneously together with the elemental Fe flux, Bi_2_Te_3_ islands are favored to form in the two ends of the dented FeTe_2_ nanorods, where the kink edges provides the most favorable sites for them to sit in, attributed to the fundamental understanding based on surface energy minimization scheme. Figure 5 is a flow chart illustrating the formation mechanisms described above.

## 4. Conclusions

The MBE-grown Bi_2_Te_3_ thin film with increasing Fe incorporation up to 6.9% in atomic concentration was found to result in two types of Fe-Te nanostructures, including FeTe_2_ nanorods embedded in the Bi_2_Te_3_ layer and a thin FeTe layer formed near the surface. A SC transition at 12.3 K of this thin film was observed in its electrical transport and its origin is revealed to be located at the interface between the thin FeTe layer and its neighboring Bi_2_Te_3_ layer. A phenomenological model about the formation mechanisms of the observed nanostructures was proposed based on the strong reaction between Fe and Te atoms. This study indicates that *in situ* heavy doping of a highly reactive dopant could result in unexpected phases of nanostructures embedded in the host matrix, providing an unusual synthesis strategy for fabricating low-dimensional nanostructured materials.

## Supplementary Materials

The following are available online at https://www.mdpi.com/2079-4991/9/5/782/s1, Figure S1: Cross-sectional TEM image showing an FeTe_2_ nanorod that its root lands inside the Bi_2_Te_3_ layer and does not reach the GaAs substrate, Figure S2: TEM images and EDS analysis of FeTe_2_ nanorod and Bi_2_Te_3_ island, Figure S3: STEM image and EDS mapping results of an area containing FeTe phase embedded in the Bi_2_Te_3_ thin film, Figure S4: Electrical transport and magnetotransport results, supporting that the SC observed in the Bi_2_Te_3_:Fe sample originates from the interface between the FeTe nanostructure and Bi_2_Te_3_.

## References

[1] Fu L., Kane C.L., Mele E.J. (2007). Topological insulators in three dimensions. Phys. Rev. Lett..

[2] Moore J.E., Balents L. (2007). Topological invariants of time-reversal-invariant band structures. Phys. Rev. B.

[3] Roy R. (2009). Topological phases and the quantum spin Hall effect in three dimensions. Phys. Rev. B.

[4] Zhang H.J., Liu C.X., Qi X.L., Dai X., Fang Z., Zhang S.C. (2009). Topological insulators in Bi_2_Se_3_, Bi_2_Te_3_ and Sb_2_Te_3_ with a single Dirac cone on the surface. Nat. Phys..

[5] Hsieh D., Qian D., Wray L., Xia Y., Hor Y.S., Cava R.J., Hasan M.Z. (2008). A topological Dirac insulator in a quantum spin Hall phase. Nature.

[6] Chen Y.L., Analytis J.G., Chu J.H., Liu Z.K., Mo S.K., Qi X.L., Zhang H.J., Lu D.H., Dai X., Fang Z. (2009). Experimental Realization of a Three-Dimensional Topological Insulator, Bi_2_Te_3_. Science.

[7] Xia Y., Qian D., Hsieh D., Wray L., Pal A., Lin H., Bansil A., Grauer D., Hor Y.S., Cava R.J. (2009). Observation of a large-gap topological-insulator class with a single Dirac cone on the surface. Nat. Phys..

[8] Hor Y.S., Williams A.J., Checkelsky J.G., Roushan P., Seo J., Xu Q., Zandbergen H.W., Yazdani A., Ong N.P., Cava R.J. (2010). Superconductivity in Cu_x_Bi_2_Se_3_ and its Implications for Pairing in the Undoped Topological Insulator. Phys. Rev. Lett..

[9] Sasaki S., Kriener M., Segawa K., Yada K., Tanaka Y., Sato M., Ando Y. (2011). Topological Superconductivity in Cu_x_Bi_2_Se_3_. Phys. Rev. Lett..

[10] Liu Z.H., Yao X., Shao J.F., Zuo M., Po L., Tan S., Zhang C.J., Zhang Y.H. (2015). Superconductivity with Topological Surface State in Sr_x_Bi_2_Se_3_. J. Am. Chem. Soc..

[11] Du G., Shao J.F., Yang X., Du Z.Y., Fang D.L., Wang J.H., Ran K.J., Wen J.S., Zhang C.J., Yang H. (2017). Drive the Dirac electrons into Cooper pairs in Sr_x_Bi_2_Se_3_. Nat. Commun..

[12] Maurya S.V.K., Neha P., Srivastava P., Patnaik S. (2015). Superconductivity by Sr intercalation in the layered topological insulator Bi_2_Se_3_. Phys. Rev. B.

[13] Fu L., Kane C.L. (2008). Superconducting proximity effect and Majorana fermions at the surface of a topological insulator. Phys. Rev. Lett..

[14] Wray L.A., Xu S.Y., Xia Y.Q., Hor Y.S., Qian D., Fedorov A.V., Lin H., Bansil A., Cava R.J., Hasan M.Z. (2010). Observation of topological order in a superconducting doped topological insulator. Nat. Phys..

[15] Chang C.Z., Wei P., Moodera J.S. (2014). Breaking time reversal symmetry in topological insulators. Mrs. Bull..

[16] Chang C.Z., Zhang J.S., Feng X., Shen J., Zhang Z.C., Guo M.H., Li K., Ou Y.B., Wei P., Wang L.L. (2013). Experimental Observation of the Quantum Anomalous Hall Effect in a Magnetic Topological Insulator. Science.

[17] He K., Ma X.C., Chen X., Lu L., Wang Y.Y., Xue Q.K. (2013). From magnetically doped topological insulator to the quantum anomalous Hall effect. Chinese Phys. B.

[18] Choi Y.H., Jo N.H., Lee K.J., Yoon J.B., You C.Y., Jung M.H. (2011). Transport and magnetic properties of Cr-, Fe-, Cu-doped topological insulators. J. Appl. Phys..

[19] Jo N.H., Lee K.J., Kim C.M., Okamoto K., Kimura A., Miyamoto K., Okuda T., Kim Y.K., Lee Z., Onimaru T. (2013). Tuning of magnetic and transport properties in Bi_2_Te_3_ by divalent Fe doping. Phys. Rev. B.

[20] He Q.L., Liu H.C., He M.Q., Lai Y.H., He H.T., Wang G., Law K.T., Lortz R., Wang J.N., Sou I.K. (2014). Two-dimensional superconductivity at the interface of a Bi_2_Te_3_/FeTe heterostructure. Nat. Commun..

[21] Manna S., Kamlapure A., Cornils L., Hanke T., Hedegaard E.M.J., Bremholm M., Iversen B.B., Hofmann P., Wiebe J., Wiesendanger R. (2017). Interfacial superconductivity in a bi-collinear antiferromagnetically ordered FeTe monolayer on a topological insulator. Nat. Commun..

[22] Zhang J.S., Chang C.Z., Zhang Z.C., Wen J., Feng X., Li K., Liu M.H., He K., Wang L.L., Chen X. (2011). Band structure engineering in (Bi_1-x_Sb_x_)_2_Te_3_ ternary topological insulators. Nat. Commun..

[23] Hsieh D., Xia Y., Qian D., Wray L., Meier F., Dil J.H., Osterwalder J., Patthey L., Fedorov A.V., Lin H. (2009). Observation of Time-Reversal-Protected Single-Dirac-Cone Topological-Insulator States in Bi_2_Te_3_ and Sb_2_Te_3_. Phys. Rev. Lett..

[24] Zhang J.M., Ming W.M., Huang Z.G., Liu G.B., Kou X.F., Fan Y.B., Wang K.L., Yao Y.G. (2013). Stability, electronic, and magnetic properties of the magnetically doped topological insulators Bi_2_Se_3_, Bi_2_Te_3_, and Sb_2_Te_3_. Phys. Rev. B.

[25] Wang Z.W., Taskin A.A., Frölich T., Braden M., Ando Y. (2016). Superconductivity in Tl_0.6_Bi_2_Te_3_ Derived from a Topological Insulator. Chem. Mater..

[26] Yavorsky B.Y., Hinsche N.F., Mertig I., Zahn P. (2011). Electronic structure and transport anisotropy of Bi_2_Te_3_ and Sb_2_Te_3_. Phys. Rev. B.

[27] Koz C., Rößler S., Tsirlin A.A., Wirth S., Schwarz U. (2013). Low-temperature phase diagram of Fe_1+y_Te studied using x-ray diffraction. Phys. Rev. B.

