# Supplementary material for: Formation of Fe-Te Nanostructures during in Situ Fe Heavy Doping of Bi2Te3
Source: Nanomaterials (Basel). 2019 May 22;9(5):782. doi: 10.3390/nano9050782 (PMC6567232; doi:10.3390/nano9050782)
Supplement: Supplementary file 1 [file nanomaterials-09-00782-s001.pdf]

Supplementary Materials: Formation of Fe-Te nanostructures during *in situ* Fe heavy doping of Bi_2Te_3

Jing Liang, Xiong Yao, Yu Jun Zhang, Fei Chen, Yuanzhen Chen and Iam Keong Sou *

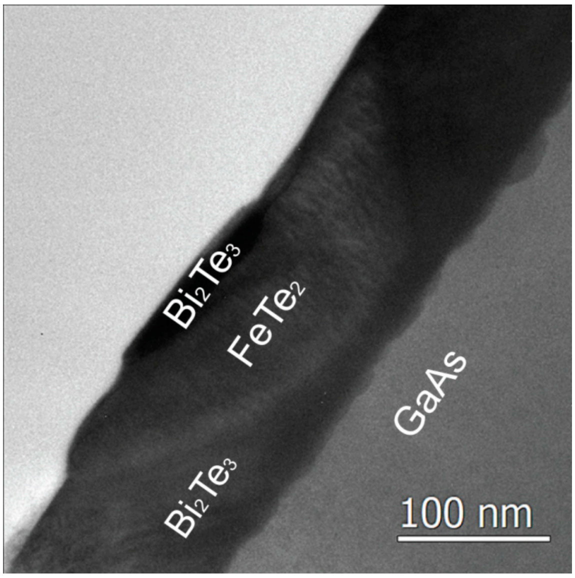

Figure S1. Cross-sectional TEM image showing an $FeTe_2$ nano-rod that its root lands inside the Bi_2Te_3 layer and does not reach the GaAs substrate.

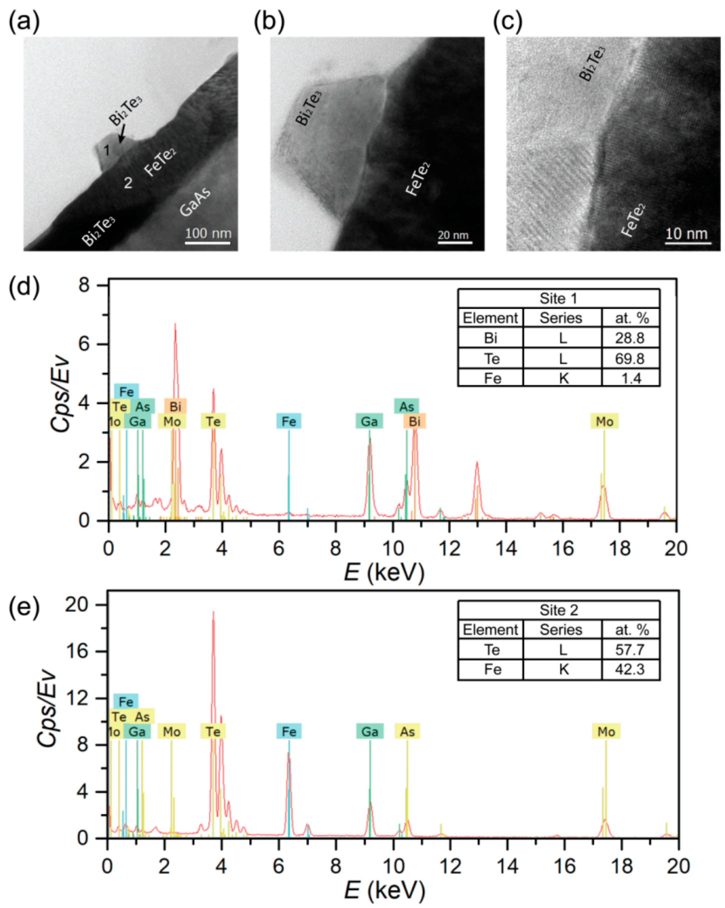

Figure S2. TEM images and EDS analysis of $FeTe_2$ nanorod and Bi_2Te_3 island. (**a**) A cross-sectional TEM image of a $FeTe_2$ nanorod with a Bi_2Te_3 island on it. (**b**) and (**c**) Zoomed-in TEM images of the boundary between the $FeTe_2$ nanorod and the Bi_2Te_3 island, showing that Bi_2Te_3 island contains several grains with different orientations. (**d**) and (**e**) EDS spectra obtained in site 1 and 2 marked in (**a**), respectively. For atomic concentration calculation, Mo signal from the sample holder, Ga and As signals from the substrate were excluded.

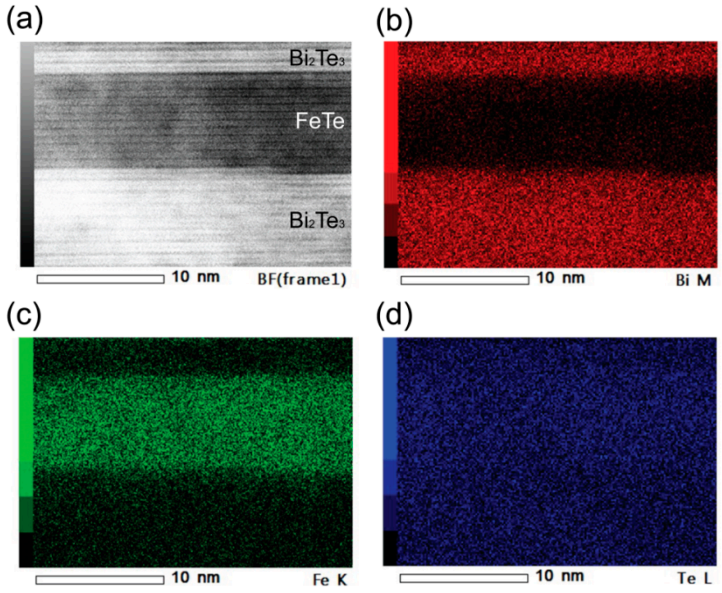

Figure 3. STEM image and EDS mapping results of an area containing FeTe phase embedded in the Bi_2Te_3 thin film. (**a**) STEM image of a FeTe nanostructure formed near the Bi_2Te_3 surface. Corresponding EDS mapping of (**b**) Bi, (**c**) Fe and (**d**) Te signals obtained from the same area in (**a**).

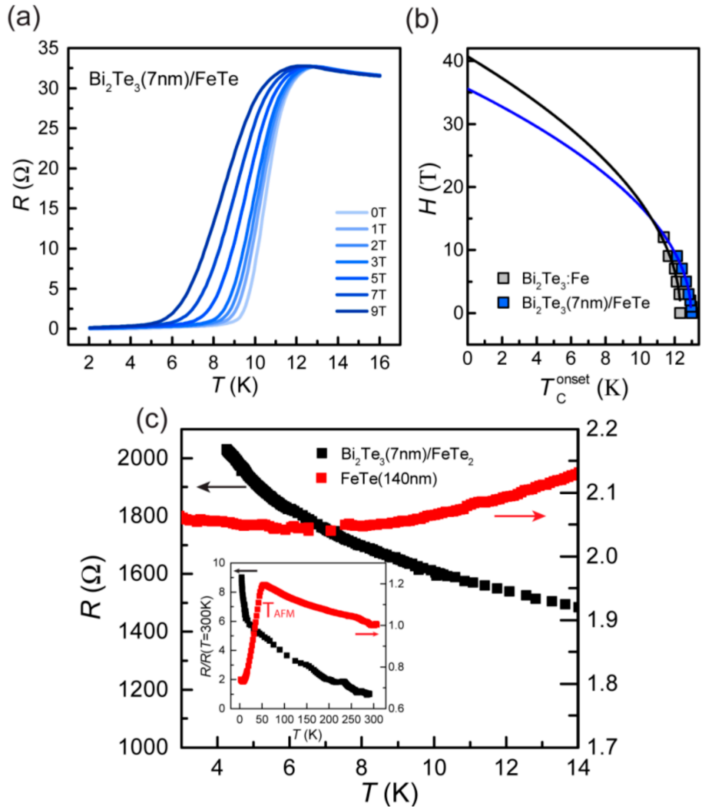

Figure S4. Electrical transport and magneto-transport results, supporting that the SC observed in the Bi_2Te_3:Fe sample originates from the interface between the FeTe nanostructure and Bi_2Te_3. (**a**) Temperature dependent resistance of a Bi_2Te_3(7 nm)/FeTe heterostructure under magnetic fields ranging from 0 to 9 T. (**b**) H_{C2} critical fields extrapolated from Ginzburg-Landau equations of the Bi_2Te_3:Fe sample and the Bi_2Te_3 (7 nm)/FeTe heterostructure are 40.7 T and 35.6 T, respectively, their similarity also acts as a further confirmation that the SC in Bi_2Te_3:Fe shares the same origin with Bi_2Te_3/FeTe. (**c**) Temperature dependent resistance of a Bi_2Te_3/$FeTe_2$ heterostructure and a pure FeTe (140 nm) thin film. Neither of them is superconducting, indicating the observed SC in Bi_2Te_3:Fe sample could only be attributed to the interface between the FeTe nanostructure and Bi_2Te_3. The Insert shows the resistance of these two samples in a wider temperature range, where one can find that the pure FeTe thin film shows an antiferromagnetic (AFM) transition at around 50 K.

© 2019 by the authors. Licensee MDPI, Basel, Switzerland. This article is an open access article distributed under the terms and conditions of the Creative Commons Attribution (CC BY) license (http://creativecommons.org/licenses/by/4.0/).
